# Fabrication of nanostructured ZnO film as a hole-conducting layer of organic photovoltaic cell

**DOI:** 10.1186/1556-276X-8-240

**Published:** 2013-05-16

**Authors:** Hyomin Kim, Yiseul Kwon, Youngson Choe

**Affiliations:** 1 Department of Chemical Engineering, Pusan National University, Busan 609-735, South Korea

**Keywords:** Electronic materials, Polymers, Vapor deposition, Sol-gel process, ZnO, Nanostructured fibrous film

## Abstract

We have investigated the effect of fibrous nanostructured ZnO film as a hole-conducting layer on the performance of polymer photovoltaic cells. By increasing the concentration of zinc acetate dihydrate, the changes of performance characteristics were evaluated. Fibrous nanostructured ZnO film was prepared by sol-gel process and annealed on a hot plate. As the concentration of zinc acetate dihydrate increased, ZnO fibrous nanostructure grew from 300 to 600 nm. The obtained ZnO nanostructured fibrous films have taken the shape of a maze-like structure and were characterized by UV-visible absorption, scanning electron microscopy, and X-ray diffraction techniques. The intensity of absorption bands in the ultraviolet region was increased with increasing precursor concentration. The X-ray diffraction studies show that the ZnO fibrous nanostructures became strongly (002)-oriented with increasing concentration of precursor. The bulk heterojunction photovoltaic cells were fabricated using poly(3-hexylthiophene-2,5-diyl) and indene-C60 bisadduct as active layer, and their electrical properties were investigated. The external quantum efficiency of the fabricated device increased with increasing precursor concentration.

## Background

Clean and renewable energy has been a considerable issue in the last decade. For this reason, organic photovoltaic cells (OPCs) have been attractive devices as next-generation substitute energy sources [1-4]. At present, the performance of OPCs has been reported up to power conversion efficiency (PCE) of 10% and above [5,6]. There have been reports that polymer solar cells have many advantages of cost effectiveness in the fabrication process, and the mechanical flexibility and polymeric materials provide a wide field of applications. Furthermore, the advantage of organic photovoltaic cells has a high potential to be manufactured using continuous coating technology capable of producing large areas at a low cost [7,8]. Poly(3,4-ethylenedioxythiophene:poly(4-styrenesulfonate)) (PEDOT:PSS) is the most widely utilized as hole-conducting layer material in organic light-emitting diodes and photovoltaic cells [9]. The advantages of PEDOT:PSS include low temperature, excellent stability, large area processing, low cost, and flexibility. However, the efficiency of this material is limited by their low carrier mobility [10]. Therefore, hole mobility is a key parameter for photovoltaic devices with respect to their adaption in device applications.

ZnO has received much attention over the past few years because of its wide range of properties that depend on doping, including a range of conductivity from metallic to insulating (including n-type and p-type conductivity), high transparency, piezoelectricity, wide-bandgap semiconductivity, room-temperature ferromagnetism, and huge magneto-optic and chemical-sensing effects. Without much effort, it can be grown into many different nanoscale forms, thus allowing various novel devices to be achieved [11]. ZnO, a II-VI semiconductor, is now recognized as a promising candidate for blue and ultraviolet light-emitting diodes or laser diodes due to its wide bandgap of 3.37 eV and large exciton binding energy of 60 meV [12-17]. Its large exciton binding energy allows excitonic absorption and recombination even at room temperature, which makes this material appealing [17]. A lot of methods have been extensively used for oriented ZnO film synthesis, including laser molecular beam epitaxy, pulsed laser deposition, metal-organic chemical vapor deposition, sputtering [12], cathodic magnetron sputtering and reactive electron beam evaporation, spray pyrolysis, and electrodeposition. However, sol-gel processes are particularly adapted to produce ZnO colloids and films in a simple, low-cost, and highly controlled way. The sol-gel process, also called soft chemistry (‘*chimie douce*’), allows elaboration of a solid material from a solution by using a sol or a gel as an intermediate step and at much lower temperatures than is possible by traditional methods of preparation [18]. It enables the powderless processing of glasses, ceramics, and thin films or fibers directly from a solution. The synthesis of solid materials via *chimie douce* often involves wet chemistry reactions and sol-gel chemistry based on the transformation of molecular precursors into an oxide network by hydrolysis and condensation reactions [19,20].

Recently, poly(3-hexylthiophene) (P3HT) has been used as a hole transporter in combination with ZnO nanostructures. These devices have an efficiency of approximately 0.5% under standard solar conditions (AM 1.5, 100 mW/cm^2^) and show a current density of *J*_sc_ = 2.2 mA/cm^2^, an open-circuit voltage of *V*_oc_ = 440 mV, and a fill factor of 0.56. This cell performance can be significantly improved to *J*_sc_ = 10.0 mA/cm^2^, *V*_oc_ = 475 mV, and a fill factor of 0.43, leading to an efficiency of 2% by using a blend of P3HT and (6,6)-phenyl-C61-butyric acid methyl ester. The low open-circuit voltage in hybrid solar cells using ZnO as the electrode material is not yet fully understood. Certainly, more investigation is necessary to find the leakage, and then higher cell efficiencies can be expected [21].

In this work, we have investigated the structural, morphological, and optical properties of ZnO nanostructured fibrous film spin coated on indium-tin oxide (ITO) glass. We fabricated polymer solar cells that have the structure of ITO/ZnO/PEDOT:PSS/active layer (P3HT:ICBA)/Al. Poly(3-hexylthiophene-2,5-diyl) (P3HT) and indene-C60 bisadduct (ICBA) were blended and used as an active layer in polymer bulk heterojunction (BHJ) photovoltaic cells. The performance characteristics of polymer photovoltaic cells using ZnO nanostructured fibrous film as a hole-conducting layer have been investigated.

## Methods

### Materials

ITO thin films are a highly degenerate n-type semiconductor which have a low electrical resistivity of 2 to 4 × 10^−4^ Ω cm. The low resistivity value of ITO film is due to high carrier concentration. It has a wide-bandgap semiconductor (3.5 to 4.3 eV), which shows high transmission in the visible wavelength (80% to 90%) and relatively high work function (4.7 eV). The ITO glass substrates were supplied from Samsung Corning Precision Materials Co. Ltd (Seoul, Korea). PEDOT:PSS aqueous solution (1.3 wt.%) as a buffer layer material was purchased from Baytron® (Hanau, Germany). Zinc acetate dihydrate as a precursor material was purchased from Junsei Chemical (Tokyo, Japan). P3HT as an electron donor and ICBA as an electron acceptor were purchased from 1-material Co. (Quebec, Canada). 1,2-Dichlorobenzene and isopropanol as a solvent were purchased from Sigma-Aldrich (Seoul, South Korea). Monoethanolamine as additive was purchased from Junsei Chemical (Tokyo, Japan).

### Preparation of ZnO nanostructured fibrous film

The pre-patterned ITO glass substrates were cleaned with acetone, ethanol, and isopropyl alcohol (1:1:1) for 1 h by sonication and then rinsed with ethanol. After cleaning, the ITO glass substrates were annealed at 230°C for 10 min in vacuum and served as high-work function electrode. ZnO nanostructured fibrous films were prepared by sol-gel process in which zinc acetate dihydrate (Zn(CH_3_COO)_2_ · 2H_2_O) was added to a solution of isopropanol and monoethanolamine. The molar ratio of zinc acetate dihydrate and monoethanolamine was 1:1, and the zinc concentration in isopropanol was set from 0.2 to 1.0 M. The mixture was stirred at 60°C for 2 h to yield a clear homogeneous solution. After stirring, the solution was spin coated at 3,000 rpm for 20 s on the pre-patterned ITO glass. The films were then dried at various temperatures for 3 h and then cooled to room temperature on a hot plate. The ZnO nanostructured fibrous films were observed under scanning electron microscopy (SEM; S-4800, Hitachi, Tokyo, Japan). The crystal structures of the samples were characterized using an X-ray diffractometer (XRD; D8 Advance, Bruker AXS GmbH, Ettlingen, Germany) with CuKa (*k* = 1.5418 Å) radiation.

### Device fabrication

PEDOT:PSS was used as a buffer layer material and filtered using a 0.45-μm Millipore polytetrafluoroethylene syringe filter (Millipore Co., Billerica, MA, USA). PEDOT:PSS was stirred for 1 h and then spin coated on the ZnO nanostructured fibrous film at 3,000 rpm for 60 s using a digitalized spin coater (MS-A10, Mikasa Co. Ltd., Tokyo, Japan). The PEDOT:PSS thin films were annealed for 20 min at 120°C in vacuum to remove the water. After the annealing process, the devices were cooled down to room temperature. The bulk heterojunction active layer was prepared via solution process. P3HT and ICBA were dissolved in 1,2-dichlorobenzene in a weight ratio of 1:1 and concentration of 20 mg/ml solution. The blend of P3HT and ICBA was stirred for 24 h at 40°C. The blend of P3HT:ICBA solution was spin coated on the PEDOT:PSS buffer layer at 2,000 rpm for 60 s. After spin coating the active layer, Al cathode was thermally evaporated onto the active layer in vacuum with a thickness of 100 nm. The thickness was measured using a well-calibrated quartz crystal thickness monitor (CRTM-600, ULVAC Kiko Co. Ltd., Saito Japan). The vacuum pressure was under 3 × 10^−5^ Torr, and the deposition rate of aluminum was controlled from 1 to 5 Å/s. The fabricated devices were subsequently post-annealed for 10 min at 150°C in vacuum condition.

## Results and discussion

### X-ray diffraction spectra

**Figure 1:**
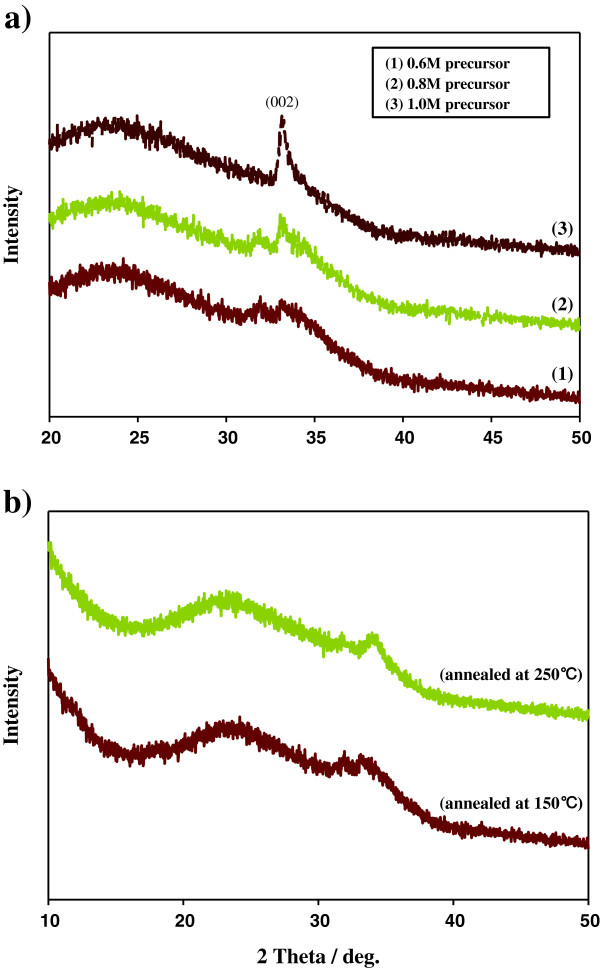
**X-ray diffraction spectra of the ZnO nanostructured fibrous films.** (**a**) With 0.6, 0.8, and 1.0 M of precursor concentration. (**b**) Synthesized at various temperatures with a concentration of 0.6 M.

The X-ray diffraction spectra of ZnO nanostructured fibrous films are shown in Figure 1. Figure 1a displays the XRD patterns of ZnO nanostructured fibrous films with different precursor concentrations of 0.6, 0.8, and 1.0 M and annealed at 150°C for 3 h. Figure 1b shows XRD patterns of films synthesized at various temperatures (150°C and 250°C). The peaks became strong with the increase in precursor concentration and drying temperature. The XRD patterns of the ZnO film had peaks assigned to ZnO (JCPDS no. 36–1451). As precursor concentration increases, the ZnO nanostructured fibrous films became strongly (002)-oriented (Figure 1a). Under the concentration of 0.6 M, we could not observe the peaks of ZnO because of the low density of the nanostructured fibrous film. Despite the same concentration (0.6 M), ZnO nanostructured fibrous films with (002) orientation were obtained depending on annealing conditions (Figure 1b). Generally, ZnO is easily ordered to (002) orientation because of low surface energy [22].

### Scanning electron microscopy

**Figure 2:**
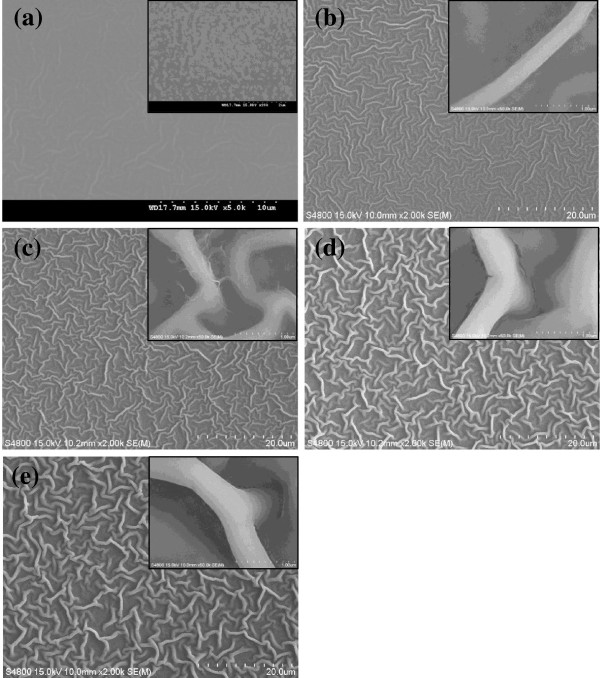
**Scanning electron microscopy of the ZnO fibrous nanostructure films on the ITO glass.** 0.2 (**a**), 0.4 (**b**), 0.6 (**c**), 0.8 (**d**), and 1.0 M (**e**) precursor.

The SEM images of the ZnO film on ITO glass are shown in Figure 2. Figure 2 shows the surface of the ZnO films, which were prepared from (a) 0.2, (b) 0.4, (c) 0.6, (d) 0.8, and (e) 1.0 M solution of zinc acetate dihydrate precursor in isopropyl alcohol and were dried on a hot plate at 150°C for 3 h and cooled slowly to room temperature. In Figure 2a, the ZnO film was not formed completely. In Figure 2b, the ZnO nanostructure was about to be formed; however, the nanostructure formed vaguely. In Figure 2c,d,e, the nanostructure of ZnO film grew clearly and thickly as the concentration of precursor increases. The grown fibrous structure had taken the shape of a maze-like structure. The increase from 300 to 600 nm of the fibrous nanostructure was observed with increasing concentration of precursor. Increase of the thickness and length of the fibrous nanostructure is relative to the increase of growth rate. As precursor concentration continues to increase, the number of Zn^2+^ and OH^−^ increases; because of that, nucleation is achieved easily, and growth rate increases at the same time. This kind of fibrous nanostructure can be formed by the possibility, that is, fibrous nanostructure is created during slow-drying condition. While during slow cooling, there is enough time for ions to aggregate along the crystal planes having similar lattice match in order to decrease their high surface energy [22]. Because fibrous nanostructures have more effective surface area than smooth surface, ZnO fibrous nanostructure is expected to be used in photovoltaic devices.

### UV-visible absorption spectra

**Figure 3:**
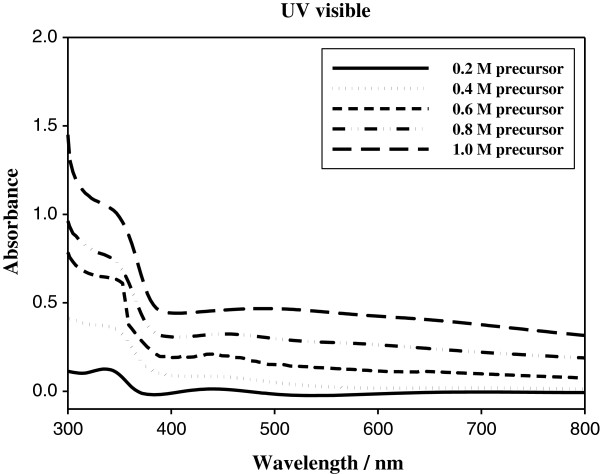
UV–vis absorption spectra of the ZnO fibrous nanostructure films with increasing concentration of precursor.

For the ZnO fibrous nanostructure films, the UV-visible absorbance spectra are shown in Figure 3. As the concentration of precursor increased, the UV-visible absorbance intensity was rapidly increased in the wavelength range of approximately 380 nm in the ultraviolet region and generally increased around all area including the visible region. Therefore, the absorbance was dependent on the concentration of the precursor. Furthermore, ZnO fibrous nanostructure films can protect light oxidation of the device by the ultraviolet area.

### Performance characteristics

**Figure 4:**
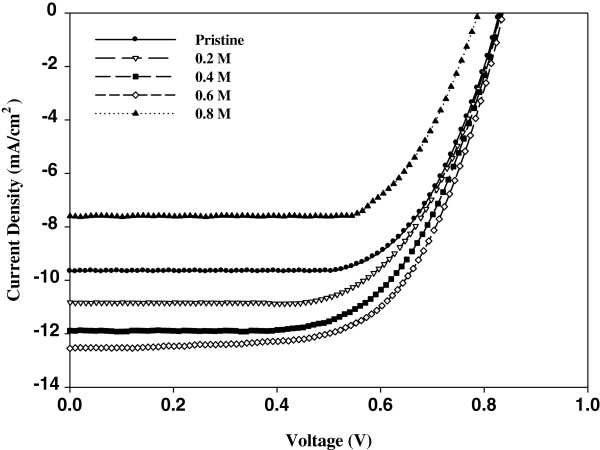
***J*-*V *curves of the devices.** ITO/PEDOT:PSS/ICBA:P3HT/Al and ITO/ZnO(0.4, 0.6, and 0.8 M precursor)/PEDOT:PSS/ICBA:P3HT/Al.

The current density-voltage (*J*-*V*) curves of the polymer solar cells are shown in Figure 4, and the data are summarized in Table 1. Polymer photovoltaic cells with the structure of ITO/ZnO fibrous nanostructure film (0.2, 0.4, 0.6, and 0.8 M precursor)/PEDOT:PSS/P3HT:ICBA (1:1 wt.%, 20 mg/ml)/Al were fabricated. Organic solar cell generates photocurrent by photovoltaic effect while passing the sunlight through the cell. That is why, using the current–voltage characteristics in the fourth quadrant at illumination in AM 1.5 conditions, we measured the typical parameters of the cells in the regime of photoelement, such as short-circuit current, open-circuit voltage, fill factor (FF), and power conversion efficiency. The pristine cell has obtained a *J*_sc_ of 8.9757 mA/cm^2^ and PCE of 4.55%. The device including ZnO fibrous film (0.6 M precursor) has a *J*_sc_ of 12.55 mA/cm^2^, and the overall PCE of 6.02% was achieved. Furthermore, *V*_oc_ was improved from 0.8286 to 0.8360 V, and PCE improved from 4.55% to 6.02%. This achievement is attributed to the advancement in the current flow and morphology result of ZnO application on the ITO. It is considered that the wide energy bandgap of ZnO may increase the mobility of holes and result in a wide effective surface area of ZnO nanofiber structures. The hole-transporting ability was improved as the applied ZnO fiber film has 3.36 eV of bandgap between the anode (ITO) and active layer (P3HT:ICBA), therefore resulting in increased *J*_sc_. However, FF of the devices decreases from 0.6124 to 0.5976 when applying the ZnO film. As the ZnO film prepared from 0.8 M Zn^2+^ precursor solution was applied to the device, there were decreases in all electrical characteristics (*V*_oc_, *J*_sc_, FF, and PCE). This phenomenon resulted from the very rough surface of the ZnO film on the ITO glass. The rough surface of the ZnO film hinders the device from making uniform photovoltaic cells. In this work, we illustrated the power conversion efficiency of 6.02% and open-circuit voltage of 12.55 mA/cm^2^ by optimizing the ZnO film through the application of 0.6 M of precursor concentration.

**Table 1:** Performance characteristics of the photovoltaic devices

**Device**	**Short-circuit current (mA/cm**^**2**^**)**	**Open-circuit voltage (V)**	**Fill factor**	**Power conversion efficiency (%)**
Pristine	8.9757	0.8286	0.6124	4.5545
0.2 M precursor	9.9191	0.8306	0.6226	5.1293
0.4 M precursor	11.4798	0.8318	0.6057	5.7841
0.6 M precursor	12.5483	0.8360	0.5976	6.0196
0.8 M Precursor	7.8613	0.7150	0.5636	3.1679

Devices: ITO/PEDOT:PSS/ICBA:P3HT/Al and ITO/ZnO (0.4, 0.6, 0.8 M precursor)/PEDOT:PSS/CBA:P3HT/Al.

### External quantum efficiency

**Figure 5:**
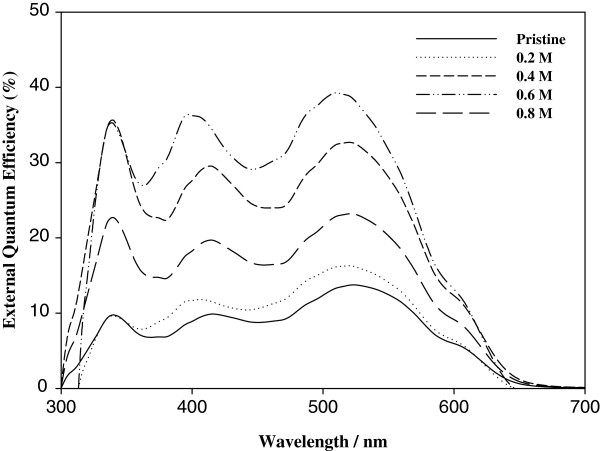
External quantum efficiency of the devices as precursor concentration increases 0.4 to 0.8 M.

External quantum efficiency (EQE) characterization of cells with the structure of ITO/ZnO film/PEDOT:PSS/P3HT:ICBA (1:1 wt.%)/Al is shown in Figure 5. When applying ZnO film with 0.2 M precursor concentration, there was no difference compared to the pristine device. There were three peaks around 340, 415, and 520 nm. For the pristine device and the device with 0.2 M precursor concentration, the maximum external quantum efficiency of 14.0% and 16.4% at 520 nm was achieved, while the PCE of the devices was 4.55% and 5.13%, respectively. In the device containing more than 0.4 and 0.6 M precursor concentration, large improvement in EQE was observed. However, a decrease of nearly half of the whole area was observed in the device including ZnO film prepared from 0.8 M of precursor concentration. It is attributed to the high surface roughness of the ZnO film. It could disrupt the fabrication of uniform photovoltaic devices. For the ZnO films prepared from 0.4 and 0.6 M of precursor concentration, a small blueshift of 415 to 400 nm and 520 to 510 nm in the photo response of the nanostructured device was observed. This blueshift in the EQE of the devices could be due to increased crystallizability of the ZnO fiber films. The ZnO film-incorporated device prepared from 0.6 M of precursor concentration achieved a maximum external quantum efficiency of 39.3% at 510 nm.

## Conclusions

In this work, we synthesized ZnO fibrous nanostructure by sol-gel process with various precursor concentrations. We have investigated the performance characteristics of organic photovoltaic cells using nanostructured ZnO film as a hole-transporting layer. ZnO film-based photovoltaic cells were focused on the dependency of Zn^2+^ precursor concentration with morphology. By adding ZnO fiber film, the conductivity and carrier mobility of the device were improved. As the precursor concentration increased, ZnO (002) orientation was observed. In a morphological aspect, with increasing concentration of precursors (0.2 to 1.0 M), the fibrous structure grew with a thickness of 300 to 600 nm and a maze-like structure. Fibrous structures have more effective surface area than smooth surface; ZnO fibrous structure is expected to be used in photovoltaic devices. For the photoluminescence aspect, the UV and green-yellow PL intensities increase with increasing concentration of precursor from 0.2 to 1.0 M. The UV-visible spectra studies show that a rapid increase of intensity at the whole wavelength area was observed. Especially, intensity at the ultraviolet area increased rapidly. The external quantum efficiency of the device was improved at the whole wavelength. The performance characteristics of polymer BHJ photovoltaic cells using ZnO fiber film as a hole-conducting layer and a P3HT:ICBA blended active layer have been investigated. As the concentration of Zn^2+^ precursors increased from 0.2 to 0.6 M, *V*_oc_, *J*_sc_, and PCE increased. This improvement can be explained by an increased charge carrier mobility of holes and electrons. However, as the concentration of Zn^2+^ precursor reached 0.8 M, all values of the characteristic parameters decreased. The polymer photovoltaic cells with the structure ITO/PEDOT:PSS (180°C for 1 h annealing)/P3HT:ICBA (20 mg/ml) (1:1 wt.%)/Al (100 nm) were investigated with the maximum power conversion efficiency of 6.02%.

## Abbreviations

BHJ: polymer bulk heterojunction; ICBA: Indene-C60 bisadduct; ITO: Indium-tin-oxide; Jsc: Short-circuit current; PEDOT:PSS: Poly(3,4-ethylenedioxythiophene:poly(4-styrenesulfonate)); P3HT: Poly(3-hexylthiophene-2,5-diyl); SEM: Scanning electron microscopy; Voc: Open-circuit voltage; XRD: X-ray diffraction.

## Competing interests

The authors declare that they have no competing interests.

## Authors’ contributions

HK conceived of the study, carried out the fabrication of photovoltaic cells, and drafted the manuscript. YK participated in estimating the photovoltaic cells and helped analyze the data. YC helped evolve the idea, guided the study, and drafted the manuscript. All authors read and approved the final manuscript.

## Authors’ information

HK and YK are MSc students at the Chemical Engineering Department, Pusan National University, South Korea. YC is a professor in the Chemical Engineering Department, Pusan National University, South Korea.
